# Multiple Roles for Chemokines in the Pathogenesis of SIV Infection

**DOI:** 10.2174/157016209787048537

**Published:** 2009-01

**Authors:** Todd A Reinhart, Shulin Qin, Yongjun Sui

**Affiliations:** 1 Department of Infectious Diseases and Microbiology, Graduate School of Public Health, University of Pittsburgh, Pittsburgh, USA; 2 Current Address: Vaccine Branch, Molecular Immunogenetics and Vaccine Research Section, National Cancer Institute, Bethesda, MD, USA

**Keywords:** SIV, HIV-1, chemokine, pathogenesis, T_reg_.

## Abstract

Chemokines are small chemoattractant cytokines involved in homeostatic and inflammatory immune cell migration. These small proteins have multiple functional properties that extend beyond their most recognized role in controlling cellular migration. The complex immunobiology of chemokines, coupled with the use of subsets of chemokine receptors as HIV-1 and SIV entry co-receptors, suggests that these immunomodulators could play important roles in the pathogenesis associated with infection by HIV-1 or SIV. This review provides an overview of the effects of pathogenic infection on chemokine expression in the SIV/macaque model system, and outlines potential mechanisms by which changes in these expression profiles could contribute to development of disease. Key challenges faced in studying chemokine function in vivo and new opportunities for further study and development of therapeutic interventions are discussed. Continued growth in our understanding of the effects of pathogenic SIV infection on chemokine expression and function and the continuing development of chemokine receptor targeted therapeutics will provide the tools and the systems necessary for future studies of the roles of chemokines in HIV-1 pathogenesis.

Chemokines are chemoattractant cytokines that are generally less than 100 amino acids in length and that play major roles in immune cell trafficking during homeostatic and inflammatory immune processes [1]. There are approximately 50 known chemokines that have been genetically and functionally grouped based on the positions of conserved cysteine residues in the amino-terminus of the mature protein [1]. These important immune factors exert their functions through members of the conserved family of seven transmembrane spanning, G-protein coupled receptors, of which approximately 20 have been shown to mediate chemokine-driven cell migration [2]. In addition to their central role in cell migration, chemokines also exhibit other activities that could contribute to or protect from pathogenesis associated with simian immunodeficiency virus (SIV) infection in non-human primates, which serves as an excellent model system for HIV-1 infection and disease. These properties include: protection from apoptosis [3, 4]; activation of pro-survival protein kinases [5, 6]; angiogenesis and anti-angiogenesis [7, 8]; chemorepulsion at high concentrations [9]; dendritic cell (DC) licensing [10]; antimicrobial activity [11]; and T cell polarization [12, 13]. Therefore, changes in chemokine expression and function could have multiple effects on immune inductive and effector activities during the course of SIV infection.

The critical role(s) played by chemokines and their receptors in HIV-1 infection and disease was poignantly revealed by the discoveries that CXCR4 [14] and CCR5 [15-19] are HIV-1 entry co-receptors. Subsequent studies revealed that nearly all SIVs use predominantly CCR5 for entry (e.g., [20-22]). The discovery that a naturally occurring deletion in CCR5 is associated with increased protection from both acquisition of HIV-1 [23-25] and subsequent disease [26] underscored the importance of chemokine receptors in HIV-1 pathogenesis. Interest in the potential for endogenous or therapeutically delivered chemokines to protect from infection fit with the clear ability of CCR5 and CXCR4 ligands to protect cells from infection *in vitro* [27-29]. Nonetheless, increased expression of CCR5 ligands *in vivo* has not proven to correlate consistently with reduced levels of viral replication in nonhuman primate models, likely due to the pro-inflammatory properties of these chemokines and their recruitment of additional cells that serve as cellular substrates for infection, thereby potentially fueling ongoing viral propagation [30]. In a similar fashion, treatment of the female reproductive tract with either TLR7 or TLR9 agonists, which would be expected to stimulate innate anti-viral responses and prevent SIV transmission, actually created a pro-inflammatory milieu that did not protect from viral transmission, likely due to recruitment of immune cells susceptible to infection [31]. Given their roles at the interface between host and pathogen, and between innate and adaptive immunity, obtaining a greater understanding of the roles played by chemokines and chemokine receptors in HIV-1/SIV infection and disease is of central importance. In this review, we focus on the broad immunobiological aspects of chemokine function during pathogenic SIV infection *in vivo* and associations with development of disease. Understanding basic aspects of chemokine immunobiology and changes caused by SIV infection will be crucial for improving immunotherapeutic and vaccination strategies for HIV-1.

## EFFECTS OF SIV INFECTION ON CHEMOKINE EXPRESSION PROFILES *IN VIVO*

Pursuant to the discoveries in 1996 that subsets of chemokine receptors serve as HIV-1 entry co-receptors, there was a strong effort to examine this aspect of virus/host interactions in the nonhuman primate model(s) for HIV-1 infection and disease. In 1997 alone there were more than 10 publications on the use of chemokine receptors for SIV entry into target cells [20-22, 32-41]. Likely driven by this new and exciting aspect of viral biology, the effects of SIV infection on chemokine expression *in vivo* began to be examined, first in PBMCs [42] and brain [43], with subsequent reports providing insight into chemokine induction in lymphoid [44], intestinal [45], and pulmonary [46] tissues. The over-riding theme from these and all subsequent studies has been that infection leads to the induction of pro-inflammatory chemokines in nearly all tissue types with variations in the composition, timing, and magnitude of induction.

### Chemokine expression in lymphoid tissues:

 Secondary lymphoid tissues are critical sites of soluble and cell-associated antigen sampling of peripheral tissues, and they are key compartments for the generation of cellular and humoral immune responses. Chemokines are major mediators of cell trafficking during immune inductive and effector activities, and changes in their expression patterns in lymphoid tissues could contribute to the pathogenesis of HIV-1 and SIV in multiple ways. Infection of rhesus (*Macaca mulatta*) or cynomolgus (*Macaca fascicularis*) macaques with pathogenic SIV leads to the induction of multiple inflammatory chemokines in lymph nodes and spleen including: CXCL8 [42]; CXCL9, CXCL10, and CXCL11 [47-52]; CCL3, CCL4, CCL5 [30, 44, 53]; CCL2 [44]; CCL19 [49, 54]; and CCL20 [55]. The increased expression of IFN-γ following SIV infection [47, 56, 57] is correlated with induction of CXCL9, CXCL10 and CXCL11, which are all CXCR3 ligands [52]. In a study of host responses to oral transmission of SIV, high CXCL9 and CXCL10 levels in lymph nodes after infection were associated with rapid progression of disease, whereas high levels of these chemokines in the oral mucosa were associated with slow progression of disease [58]. Induction of CCL19 could be associated with increased recruitment of mature DCs [54, 59, 60], in which CCL19 is highly expressed [61]. A number of studies (e.g., [62-64]) have examined changes in chemokine expression in peripheral blood mononuclear cells (PBMCs) from SIV or SHIV infected nonhuman primates and have found CCL3, CCL4, and CCL5 to be upregulated. One study has found that infection with SHIV can lead to decreased levels of CCL3, CCL4, and CCL5 in PBMCs during the acute phase of infection [65]. The implications of changes in chemokine expression in PBMCs on recruitment of cells to lymphoid or other tissues remain unclear.

Less completely explored yet likely important nonetheless are chemokines reduced in expression following SIV infection. Chemokines down-regulated during SIV infection include the homeostatic lymphoid chemokine CCL21 [52, 54], the Th2 recruiting chemokines CCL17 and CCL22 [52], and the anti-apoptotic chemokines CCL25 and CXCL12 (our unpublished observations). As with the induction of CXCR3 ligand expression, the reduction in CCR4 ligand expression (i.e., CCL17 and CCL22) is likely driven by increased IFN-γ [52].

The overall implications of the findings with lymphoid tissues from SIV-infected macaques suggest that SIV infection leads to development of a polarized, inflammatory milieu. These modified environments in lymphoid tissues are polarized toward type 1, IFN-γ production due to increased recruitment of T cells expressing CCR5 and CXCR3, which are found predominantly on type 1 T cells [66, 67], and due to decreased recruitment of T cells expressing CCR4, which is found predominantly on type 2 T cells and T_reg_ [68, 69]. Although it would be reasonable to expect that increased local expression of CCR5 ligands would decrease viral replication, this does not appear to be the case in these dynamic tissues [30], most likely due to the recruitment of cells susceptible to infection.

### Chemokine expression in lung tissues:

In addition to lymphoid tissues, pulmonary tissues are affected directly and indirectly by HIV-1/SIV infection. Prior to the widespread use of antiretroviral therapy (ART), at least in western Europe and North America, approximately 75 to 85% of patients with AIDS were developing some form of pulmonary disease complication [70]. With the greater use of ART, however, there has been a decline in the proportions of HIV-infected individuals in the US that develop and are hospitalized for pulmonary complications, although ART itself can lead to pulmonary complications [71-73]. SIV-infected macaques can develop common pulmonary outcomes found in HIV-1 infected humans, including *Pneumocystis carinii* pneumonia (PCP) (caused by what is now called *Pneumocystis jiroveci* in humans) and lymphoid interstitial pneumonitis (LIP) [74-78]. During pathogenic SIV infection CCL5 expression is increased in bronchoalveolar lavage mononuclear cells [46, 53]. In addition, CCL2 and CXCL10 levels are increased in lung tissues during SHIV infection, and are associated with the development of pneumonia [79]. Similarly, CXCL9, CXCL10, CXCL11, CCL2, CCL3, CCL4, and CCL5 are increased in expression in lung tissues [80] and draining lymph nodes [51] during late stages of SIV infection especially when local burdens of *P. carinii* are high (our unpublished findings).

### Chemokine expression in intestinal tissues:

Intestinal tissues have become recognized as a major target for HIV-1/SIV viral replication early after infection [81, 82] and a site of early and profound T cell depletion [45, 83-85]. Intestinal tissues also exhibit altered chemokine expression profiles due to SIV infection, consisting primarily of increased levels of inflammatory chemokines. Chemokines associated with the trafficking of DC precursors (i.e., CCL20), and mature DC, naïve T cells and central memory T cells (i.e., CCL21), do not change significantly in intestinal tissues throughout the course of infection [86]. In contrast, the CCR5 ligand CCL5 is increased in intestinal tissues during SIV infection [87], whereas CCL4 expression is decreased in jejunal intraepithelial lymphocytes (IEL) compared to uninfected animals [45]. In addition, the CXCR3 ligand CXCL9, and the CCR7 ligand CCL19, both of which can contribute to tissue inflammation, were found to be upregulated in intestinal lamina propria early after SIV infection, potentially contributing to increased homing of T cells to this compartment [49]. Interestingly, the HIV-1 gp120 was recently demonstrated to bind to the α4β7 integrin, which is a major homing receptor on gut homing T cells [88], and could therefore reduce the trafficking of T cells to the intestines.

### Chemokine expression in brain:

 Although the use of ART has reduced the incidence of HIV-associated dementia (HAD), the prevalence of HAD has not decreased because HIV-1 infected individuals are living longer [89]. HIV-1 and SIV enter CNS tissues early and can productively replicate [90-92], although neurocognitive manifestations arise late during the course of infection [93]. In a large study of encephalitic brain tissues from SIV-infected macaques, the inflammatory chemokines CCL3, CCL4, CCL5, CCL7, and CXCL10 were all shown to be elevated compared to non-encephalitic tissues and to co-localize with sites of inflammatory infiltrate [43]. Expression of CX3CL1 is also increased in encephalitic brain tissues from SIV-infected macaques [94], and microglia cultured from SIV-infected macaques express higher levels of CXCL8 than microglia from uninfected animals [42]. In addition, induction of the inflammatory chemokines CXCL10 and CCL2 was observed in encephalitic brain tissues from SHIV infected macaques [95], and increased CCL2 was present in cerebrospinal fluid of SIV-infected macaques [96]. The increased expression of these chemokines contributes to the perivascular and nodular inflammation that defines encephalitis (e.g., [43]) and could contribute to apoptosis, as CXCL10 has pro-apoptotic effects on cultured neuronal cells [97].

### Potential mechanisms leading to altered chemokine expression:

 The forces driving changes in chemokine expression in tissues during SIV infection are not fully understood although there are host and viral elements that provide insight into these mechanisms. Virion components and viral proteins can contribute to induction of pro-inflammatory mediators, including chemokines. For example, HIV-1 RNAs are recognized by TLR7 and TLR8 [98], and can induce IFN-γ production by natural killer (NK) cells [99]. In addition, increased expression of TLR3 positively correlates with increased CXCL10 expression in lymphoid tissues during SIV infection, suggesting that recognition of dsRNA intermediates of SIV replication might stimulate pro-inflammatory host responses [50]. Furthermore, HIV-1 Nef and Tat induce chemokine expression from multiple cell types including macrophages, astrocytes, T cells, and DCs [100-105]. In parallel, indirect mechanisms involving host cellular responses including type I and type II IFN production by cells of the innate and/or adaptive immune systems, will stimulate expression of pro-inflammatory chemokines such as the IFN-γ-inducible CXCR3 ligands CXCL9, CXCL10, and CXCL11, which are consistently up-regulated in multiple tissue types [43, 47, 49, 80, 95].

## POTENTIAL ROLES FOR CHEMOKINES IN SIV PATHOGENESIS

The mechanisms by which chemokines that are constitutively expressed and those that are increased or decreased in expression contribute to the pathogenesis of SIV infection are not fully understood. Nevertheless the extent of understanding continues to grow along with the understanding of the diverse functional activities of chemokines beyond their roles in orchestrating cellular migration. It is likely that the combined activities of multiple chemokines contribute to the cumulative loss of immune function during HIV-1/SIV infection, and therefore interventions that target the cell migratory and other functions of chemokines and their receptors might provide new therapeutic strategies for HIV-1 infection and disease.

### Viral entry co-receptor blockade:

 Chemokines could potentially limit the pathogenesis of HIV-1/SIV infection by blocking entry co-receptors and thereby inhibit ongoing viral propagation. The ligands for CCR5 and CXCR4, the major co-receptors for HIV-1 and SIV infection, inhibit viral replication in *in vitro* model systems [27, 29, 106, 107], and increased levels of their expression were expected to limit local levels of viral replication. However, this was not supported when examined in SIVmac239 challenged macaques previously infected (or immunized) with a non-pathogenic strain of SHIV [30]. These investigators found greater levels of SIV replication in lymphoid tissues that contained high levels of the CCR5 ligands CCL3 and CCL4 [30]. This was surprising given the low levels of transmission of HIV-1 to individuals with a 32 base pair deletion in their *ccr5* gene [23-25] and the reduced rate of disease progression in those individuals with this deletion who actually become infected [26]. In addition to lymphoid tissues, increased expression of CCR5 ligands is associated with higher lung viral RNA levels, indicating that the local expression of these chemokines was not sufficient to inhibit SIV replication in lung and lymphoid tissues [80] (and our unpublished observations). In contrast, there are data suggesting that some immunization strategies can lead to the induction of CCR5 ligands in lymphoid tissues and thereby contribute to vaccine efficacy [108-110], although not all studies have supported this view [111], perhaps due to the potential inflammatory recruitment of cells bearing CCR5 which could serve as cellular substrates for ongoing viral replication.

### Inflammatory recruitment of cellular substrates for infection:

 In addition to the competing outcomes of increased CCR5 ligand expression, other inflammatory chemokines could also impact SIV pathogenesis through the recruitment of cellular substrates for infection to sites of viral replication. The CXCR3 ligands CXCL9, CXCL10, and CXCL11 are potent chemotactic factors for activated T cells [112], which are highly susceptible to infection *in vivo* [113], and these IFN-γ-inducible chemokines are consistently increased in expression in multiple tissues following SIV infection [47, 49, 52, 80]. Thus, these chemokines likely recruit activated T cells to inflammatory tissues and provide new substrates for viral propagation. In addition, inflammatory chemokines could have a direct effect on T cells or macrophages and stimulate viral replication, as has been shown for CXCL10 [114]. Preventing migration of these cells through inhibition of chemokine or chemokine receptor function might reduce the inflammation-mediated propagation of virus.

### Changes in DC trafficking:

 Changes in chemokine expression and function due to HIV-1/SIV infection could also modulate immune function by altering the trafficking of immune cells to immune inductive sites such as draining lymph nodes. Chemokines are major players in the movement of DC precursors to peripheral sites of antigen acquisition and from there to draining lymph nodes [115]. DCs are potent antigen-presenting cells and their numbers, phenotypes and localization within lymphoid tissues are important for generating immune responses [116]. After antigen uptake and activation in peripheral tissues, DCs undergo a maturation program that includes increased expression of CCR7, which provides chemotactic responsiveness to CCL21 and draws them into the draining lymphatics [117], as well as fine-tunes their localization within the T cell rich paracortical regions of lymph nodes [118]. DC numbers in lymphoid tissues increase early during the course of HIV-1 and SIV infection and decrease during AIDS [54, 60, 119], and changes in the expression of chemokines within lymphoid tissues likely contributes to these alterations. For example, the homeostatic chemokine and CCR7 ligand, CCL21, is reduced in expression in lymph nodes in SIV-infected rhesus and cynomolgus macaques [52, 54], and its expression pattern is altered in spleen tissues by SIV infection [54]. In addition, CCR7 ligands provide licensing function to DCs [10]. Although not examined in detail, another homeostatic lymphoid chemokine expressed in germinal centers, CXCL13, is upregulated upon SIV infection [47], and might alter the trafficking of DCs, B cells, and follicular helper T cells to germinal centers [120] and thereby affect antibody responses to infectious agents. Therefore, changes in chemokine expression might contribute to immune dysfunction by altering DC numbers, phenotypes, and/or localization in lymphoid tissues.

### Polarization of immune environments:

 Changes in chemokine expression in tissues due to SIV infection could lead to the polarization of local immune environments through two potential mechanisms. First, chemokines could control the selective recruitment of T cells programmed to express specific cytokines. In addition, more systemic polarization of immune responses might arise due to the ability of chemokines to participate directly in shaping T cell differentiation.

T cell differentiation during antigenic stimulation of naïve T cells is a critical aspect of host immune responses to infection and can affect the outcome of an infection. It is clear that in addition to the original dichotomous model of T cell differentiation into IFN-γ producing type 1 cells or IL-4/IL-5 producing type 2 cells [121, 122], naïve T cells can also by shaped into inflammatory IL-17 producers (Th17) or immunosuppressive regulatory T cells (T_reg_) [123]. This shaping is influenced in large part by cytokines that are present during T cell stimulation, which in turn control the expression of lineage-specific transcription factors [123]. For example, IL-12 and IFN-γ drive differentiation into type 1 T cells, whereas IL-2 and TGF-( drive differentiation into inducible T_reg_. Although not absolute, overall chemokine receptor expression patterns are different on T cells of the different lineages. Type 1 T cells more selectively express CXCR3 and CCR5, type 2 T cells more selectively express CCR3, CCR4 and CCR8 [66-68, 124], and Th17 cells have recently been described as selectively expressing CCR6 [125]. In addition, CCR4 and CCR7 are amongst the most abundantly expressed chemokine receptors on T_reg_ [69]. Therefore, chemokines can control the cytokine polarization of local environments through the recruitment of specific subsets of T cells. The consistently observed induction in expression of CXCR3 and CCR5 ligands, and reduction in CCR4 and CCR7 ligands in lymphoid and other tissues during SIV infection, would be expected to impact the local cytokine environment by increasing the recruitment of type 1 T cells and by decreasing the recruitment of type 2 T cells and T_reg_. The induction of CXCR3 ligands will also impact the population of T cells entering tissues because they are natural antagonists for CCR3 [126, 127] and CCR4 [52], further contributing to immune polarization in local environments. The increased levels of IFN-γ present in lymphoid and other tissues during SIV infection [47, 52, 57] are consistent with this model. In turn, the increased local levels of IFN-γ will further induce CXCR3 ligand expression and reduce CCR4 ligand expression, sustaining a chemokine expression pattern that will selectively recruit type 1 T cells in an IFN-γ-driven positive feedback loop. In immune inductive sites such as lymph nodes, this polarized cytokine milieu will create an environment that favors the differentiation of naïve T cells into type 1 T cells due to the local predominance of IFN-γ.

The increased expression of subsets of chemokines could also contribute to immune polarization through their direct action on T cells during stimulation. Although this is an area of study that requires further attention, there are examples for chemokines contributing to T cell differentiation pathways. For example, the CXCR3 ligand CXCL10, which is up-regulated in lymphoid tissues during SIV infection [47, 52], will push T cells toward a type 1 phenotype when present during T cell stimulation [12]. A number of other chemokines have been shown to also affect T cell differentiation [13]. Therefore, the up-regulation of CXCR3 ligand levels in lymphoid tissues during SIV infection, and the down-regulation of CCR4 ligands [52], could further contribute to the immune polarization of local environments and type 1, inflammatory positive feedback loops.

Related to the cytokine polarization of lymphoid tissue environments, but also directly relevant to the levels of cellular activation, and possibly cell death, is the extent to which T_reg_ are present and providing a balancing, immunosuppressive force [128, 129]. The balance of T_reg_ numbers in lymphoid tissues during HIV-1/SIV infection could modulate local levels of immune activation and/or immune suppression. It is not clear whether HIV-1 or SIV definitively increase or decrease levels of T_reg_ in lymphoid tissues, although there are findings that support a loss of T_reg_ [52, 130-132] and others that support expansion of T_reg_ [133-135] during pathogenic infections. Chemokines could control these outcomes, given the preferential expression of subsets of chemokine receptors on Treg [69] such as CCR4, the loss of CCR4 ligand expression in lymphoid tissues during SIV infection [52], and antagonism of CCR4 by simultaneously up-regulated CXCR3 ligands [52].

### Protection from apoptosis:

 Increasing evidence indicates that a number of chemokines posses anti-apoptotic properties. For example, CXCL12 enhances survival and prevents apoptosis of murine embryonic [136] and bone marrow [3] stem cells, and when not proteolytically processed it also serves as a neuronal survival factor *in vitro* [137]. CXCL12 also promotes survival of CD4+ T cells by post-translational inactivation of components of the apoptotic pathways and transcriptional up-regulation of genes that support cell survival [138]. In addition, CCL25 can protect malignant T cells from chemotherapy-induced apoptosis [139], and CCL25 stimulation through CCR9 can trigger a cell survival signal that involves the survival-associated protein kinase B (also known as Akt) [140]. Furthermore, CCL21 is an antiapoptotic factor for mesangial cells [141], DCs [6], and T cells [5]. All three of these chemokines are reduced in expression in lymphoid tissues during SIV infection [52, 54], (and our unpublished observations), and could therefore contribute to increased apoptosis.

## CONSIDERATIONS IN STUYDING THE ROLES OF CHEMOKINES IN SIV IMMUNOPATHOGENESIS

Expression profiling to measure changes associated with viral infections has proven valuable in revealing potential mechanisms of viral pathogenesis and will undoubtedly continue to do so. Nonetheless, there are aspects to chemokine biology that need to be considered within a broader systems biological view when reconstructing mechanistic models of chemokine-associated pathogeneses. We describe here three of these aspects of chemokine biology.

### Nonlinear relationships between chemokine concentration and activity:

 A fundamental aspect of chemokine biology requiring consideration goes beyond the need to clarify the relationship between mRNA and cognate protein expression and the fact that chemokines are secreted proteins. Dose response studies *in vitro* have made clear that as higher concentrations of chemokines are provided to responsive cells, a maximum chemotactic response is reached after which increasing concentrations of chemokine actually lead to reduced cell migration (e.g., [142]). This means that exceptionally high local concentrations of chemokine could effectively exclude otherwise response cells. One example of this migratory behavior of cells was observed with time-lapse photomicrography and showed migration of CXCR4+ cells away from a source of CXCL12 [9].

### Posttranslational processing of chemokines:

During the last few years, proteolytic processing has become recognized as a mechanism that regulates the biological activities of many chemokines. Proteases that process chemokines include CD26, CD13, and matrix metalloproteinases (MMPs).

CD26, also known as dipeptidyl peptidase IV (DPP IV) cleaves amino-terminal X-pro dipeptides from selected proteins [143]. Approximately one-third of human chemokines has an X-pro amino-terminal dipeptide motif and therefore could be cleaved by CD26. Chemokines known to be processed by CD26 include: CXCL6 [144]; CXCL12 [145]; CCL3L1 [146]; CCL5 [144, 147, 148]; CCL11 [149]; and CXCL9, CXCL10 and CXCL11 [150, 151]. Cleavage by CD26 can have differing effects on chemokine activity depending on the chemokine, including no effect, increased agonist activity, or conversion to an antagonist [152]. The CXCR3 ligands CXCL9, CXCL10, CXCL11, which are consistently induced in multiple tissues following SIV infection, are substrates for CD26 and cleavage of these inflammatory chemokines decreases their chemotactic activity more than 10-fold and converts them into potent CXCR3 antagonists [150, 151]. Interestingly, HIV-1 Tat protein has been shown to bind CD26 [153]. In addition, HIV-1 gp120 blocks binding of adenosine deaminase to CD26 [154] and although its use as an entry co-receptor has been controversial (e.g., [155-158]), gp120 binding might modulate CD26 activity and hence the local concentrations of unprocessed CXCR3- and other ligands.

The metalloprotease aminopeptidase N (APN), also known as CD13, was recently reported to proteolytically process CXCL11 [159]. APN/CD13 is a 150 kDa membrane-bound protease that is widely expressed and has a broad functional repertoire [160]. CD13 proteolytic processing of CXCL11 into multiple amino-terminally truncated products led to reduced signaling and chemotaxis through CXCR3 and CXCR7 [159].

Matrix metalloproteinases (MMPs) are a family of proteolytic enzymes that perform multiple roles in normal immune responses to infection. Some MMPs are able to proteolytically processes subsets of chemokines, and as with CD26 processing, these modifications can increase or reduce their bioactivities [161-163]. Examples of chemokines processed by MMPs include: the monocyte chemoattractant proteins (MCPs), CCL2/MCP-1, CCL7/MCP-2, CCL8/MCP-3, and CCL13/MCP-4 [164]; CXCL8 [164]; and CXCL12 [137, 164, 165].

### Receptor antagonism by chemokines:

 In addition to their agonist properties on subsets of chemokine receptors, numerous chemokines also antagonize heterologous chemokine receptors and increased expression of chemokines during SIV infection, such as for CXCR3 ligand CXCL11, could lead to exclusion of cells from local microenvironments. Examples of this interesting cross-regulatory property of chemokines include: CXCL9, CXCL10, and CXCL11 antagonism of CCR3 [126, 127]; CXCL11 antagonism of CCR5 [166]; CCL11 antagonism of CXCR3 [167] and of CCR2 and CCR5 [168]; CCL7 antagonism of CCR5 [169]; and CCL26 antagonism of CCR2 [170] and of CCR1 and CCR5 [171]. In addition, CXCL11 antagonizes CCR4 [52]. Chemokine receptor antagonism by chemokines induced during SIV infection could contribute to the reshaping of immune cell composition and cytokine milieu in lymphoid and other tissues and exclude cells critical to balancing local and systemic immune responses. For example, increased expression of IFN-γ-inducible CXCL9, CXCL10, and CXCL11 is highly negatively correlated with CCR4 levels in lymphoid tissues of cynomolgus macaques during SIV infection [52], possibly contributing to loss of T_reg_ and increased levels of cellular activation.

## FUTURE DIRECTIONS FOR CHEMOKINE RESEARCH IN THE AREA HIV-1/SIV PATHOGENESIS

Chemokines and their receptors are clearly at the interface between the host and HIV-1/SIV in multiple regards, including viral transmission, viral propagation and chronicity, innate immunity, adaptive immunity, the interface between innate and adaptive immunity, inflammation, and cytokine milieus in immune inductive and effector sites. Additional investigation is necessary to understand the full extent and repertoire of changes to chemokine expression profiles in different tissue compartments within infected hosts. These findings need to be considered within a broader systems biological view that considers not just one chemokine and its receptor(s) and not just the chemoattractant outcomes of this interaction, but considers multiple chemokines and receptors, cross-agonism and cross-antagonism of receptors by multiple chemokines, post-translational modifications to chemokines, and the growing list of functional activities of chemokines beyond control of cell migration. Future studies will need to consider simultaneously the events occurring in multiple tissues and will need to include more sophisticated cell trafficking and homing studies. Coupling these investigations with the intense efforts being directed at developing therapeutic strategies against chemokine receptors could provide new avenues for treatment of HIV-1 infected individuals, and the SIV/macaque model system will continue to provide a robust preclinical model with which to begin to test such strategies.

Targeting chemokine receptors in the context of HIV-1 infection and disease can be viewed from multiple perspectives. The first is viral entry and replication and CCR5 antagonists have been developed and are being tested. Another view would be to target the pro-inflammatory effects of increased expression of inflammatory chemokines. Any strategy to intervene in such a way is complicated by the overlapping specificities in the chemokine system [1]. Targeting a chemokine or chemokines will leave the receptor available to be acted upon by other chemokines or could require targeting multiple chemokines that act on the same receptor. Targeting the receptor will leave the chemokines available to act on other receptors as either agonistics or antagonists. Potentially further complicating the dynamics of targeting chemokine receptors is that it has recently been shown in chemokine receptor knockout mice that this genetic and functional deletion allows greatly increased levels of the cognate chemokine to circulate systemically, which in turn can bind and down-modulate and/or desensitize other chemokine receptors [172].

The inhibition of viral entry and replication when susceptible cells are treated with entry co-receptor chemokine ligands (e.g., [27, 29, 106, 107]) provided a basis for developing modified CCR5 ligands as therapeutic approaches. CCR5 is clearly an excellent target to inhibit the transmission and subsequent replication of HIV-1, and the general lack of negative consequences to being homozygous for the Δ32 mutation in *ccr5* suggests that pharmacologic inhibition of the receptor will not lead to severe complications, which has been supported in human trials [173]. One approach has been to modify CCL5 (also known as RANTES), either by the placement of a methionine (Met-RANTES [174]) or synthetic modified analogues (AOP-RANTES [175] and PSC-RANTES [176]) at the amino-terminus of the otherwise mature protein. These CCR5 antagonists exhibit potent viral inhibitory properties that act by not only sterically blocking receptor availability to the virus, but also by inducing receptor down-modulation [177, 178]. There are also small molecule inhibitors of CCR5 that have been and are being tested in humans and nonhuman primates, including CMPD167 [179], and maraviroc [173]. Small molecule and amino-terminally modified CCL5s have been shown to be efficacious in reducing SIV replication and blocking viral transmission in nonhuman primate models [179-181]. The CCR5 inhibitor maraviroc is currently in a phase III trial in humans [173].

Manipulation of the chemokine and chemokine receptor system beyond inhibition of viral replication through CCR5 could provide tremendous insight into the contributions of pro-inflammatory chemokines to HIV-1/SIV pathogenesis. Given the consistent observation that CXCR3 ligands are up-regulated in multiple tissues during SIV infection, it would be interesting to inhibit CXCR3 in an attempt to reduce the inflammatory positive feedback loop of host IFN-γ responses, IFN-γ induction of CXCR3 ligands, and CXCR3-mediated recruitment of IFN-γ producing cells [47]. CXCR3 inhibitors have been developed and examined in clinical trials for chronic inflammatory diseases such as psoriasis and rheumatoid arthritis [182]. Similarly, manipulation of the CCL17/CCL22/CCR4 axis would allow more direct examination as to whether reducing T_reg_ levels in tissues of SIV infected macaques will reduce or exacerbate disease progression [52, 133, 134].

Progress continues to be made in understanding the multiple roles of chemokines in the pathogenesis of or protection from disease caused by HIV-1/SIV infection. Linking basic studies of the changes caused by infection, with basic studies of chemokine immunobiology and clinicopharmacologic studies of chemokine and chemokine receptors, will provide new opportunities to test hypotheses and develop novel therapeutic interventions. As with any immunologically-targeted intervention, the challenge will be to identify the optimal potency and specificity that suppresses that which is pathologic yet maintains that which is necessary for appropriate immune function.
